# Oliguria as a diagnostic marker of severe leptospirosis: a study from the Transcarpathian region of Ukraine

**DOI:** 10.3389/fcimb.2024.1467915

**Published:** 2024-10-31

**Authors:** Pavlo Petakh, Oleksandr Kamyshnyi

**Affiliations:** ^1^ Department of Biochemistry and Pharmacology, Uzhhorod National University, Uzhhorod, Ukraine; ^2^ Department of Microbiology, Virology, and Immunology, I. Horbachevsky Ternopil National Medical University, Ternopil, Ukraine

**Keywords:** mortality association, oliguria, acute kidney injury, leptospirosis, predictor

## Abstract

Leptospirosis is an emerging illness presenting a broad range of clinical manifestations, ranging from asymptomatic or mild cases to severe and fatal outcomes. Early detection is crucial for effective treatment; however, similar clinical presentations in various febrile illnesses or co-infections, along with challenges in laboratory diagnostics, can lead to misdiagnosis and severe consequences. Identifying clinical predictors for severe forms of the disease is essential in mitigating complications and reducing mortality. Consequently, we conducted a retrospective case-control study to identify clinical markers indicative of severe disease in leptospirosis patients from the Transcarpathian region. The study focused on patients admitted with clinically suspected leptospirosis, involving a total of 51 diagnosed cases, with 13 resulting in severe outcomes and death. Categorical variables were analyzed using χ^2^, revealing a mean patient age of 50 years, predominantly male (n = 36, 70.5%). Oliguria emerged as a significant independent factor associated with mortality (odds ratio [OR], 13.5; 95% confidence interval [CI], 2.56–71.12; p = 0.001). Additionally, our analysis uncovered a noteworthy increase in leptospirosis notification rates in Transcarpathian compared to Ukraine, with 150 cases out of the total 433 in Ukraine. The highest notification rates were observed in Mukachevo District and Perechyn District. These findings highlight the importance of early recognition of key clinical markers, such as oliguria, which are critical for predicting severe outcomes in leptospirosis patients. The higher notification rates in Transcarpathian regions also underscore the need for enhanced surveillance, targeted public health interventions, and timely treatment to reduce mortality in endemic areas.

## 1 Introduction

Leptospirosis represents a re-emerging zoonotic disease with a global presence, spanning all continents except Antarctica (Ullmann and Langoni, 2011; Petakh and Nykyforuk, 2022; Petakh et al., 2022a; Petakh and Kamyshnyi, 2023). Its clinical manifestations range from mild to severe, posing life-threatening consequences. Symptoms often mimic those of other infectious diseases, such as influenza (Philip et al., 2021). Severe cases, affecting approximately 5% to 15% of individuals, manifest with acute renal failure, acute respiratory distress syndrome, pulmonary complications, hypotension, icterus, and altered mental status (Panaphut et al., 2002; Doudier et al., 2006; Spichler et al., 2008; Petakh et al., 2022c; Petakh et al., 2022b). A 2015 systematic review estimated that around one million cases of leptospirosis occur globally each year, resulting in approximately 60,000 deaths (Costa et al., 2015). Between 2010 and 2021, 23 EU countries reported 12,180 confirmed cases of leptospirosis, with an average annual rate of 0.24 per 100,000 population. Five countries—France, Germany, the Netherlands, Portugal, and Romania—accounted for 79% of cases. Slovenia had the highest rate at 0.82 per 100,000 (Beauté et al., 2024). In Ukraine, 4,873 cases were reported from 2009 to 2023, with an average annual rate of 0.75 per 100,000, among the highest in Europe (Petakh et al., 2024a; Petakh et al., 2024b).

In the Transcarpathian region, leptospirosis persists as a prevalent zoonotic disease. Between 2005 and 2015, 420 cases were reported, with an incidence three times higher than the national average. The case fatality rate (CFR) for leptospirosis in Transcarpathian region averages 12.5%, exceeding the national level of 9.8% (Markovych et al., 2019; Petakh et al., 2023b; Petakh et al., 2023a).

Geographical variations worldwide contribute to distinct presentations of leptospirosis. Divergent Leptospira species and serovars, coupled with socioeconomic and environmental factors, play pivotal roles in shaping the clinical presentation of the disease (Spichler et al., 2008). Proposed variations in intrinsic virulence among serovars and species may explain differences in disease severity (Philip et al., 2021).

The spectrum of leptospirosis, ranging from mild and subclinical to severe or fatal illness, underscores the importance of vigilant patient monitoring. Identifying prognostic markers is crucial for continuous surveillance, yet the factors predicting the transition from mild to severe/fatal illness remain unclear (Dupont et al., 1997; Spichler et al., 2008).

Despite extensive research on leptospirosis, there remains a significant gap in identifying specific clinical and epidemiological predictors that signal the progression from mild to severe forms of the disease. While geographic variations and differences in Leptospira species have been studied, the factors driving severe outcomes in leptospirosis—particularly in high-incidence regions like Transcarpathia—are still poorly understood. This study aims to address this gap by examining potential predictors of severe illness, which could improve early diagnosis and intervention, thereby reducing the associated morbidity and mortality.

## 2 Materials and methods

A retrospective case-control study was conducted at the Transcarpathian Regional Clinical Infectious Diseases Hospital, involving the review of 51 medical records of patients admitted between 2012 and 2018. The study aimed to investigate factors associated with severe outcomes of leptospirosis. To avoid selection bias, only patients meeting the laboratory-confirmed diagnostic criteria for leptospirosis were included. These criteria involved positive PCR or Microagglutination Testing (MAT), in conjunction with clinical symptoms indicative of the disease. Patients without laboratory confirmation were excluded to ensure diagnostic consistency across the sample.

A standardized questionnaire was employed to collect data on patient demographics (age and gender) and clinical symptoms, including fever (≥38 °C), myalgia, jaundice, arthralgia, oliguria, nausea, vomiting, headache, and abdominal pain. Oliguria was defined as urine production of less than 400 ml per day. The age range of the included patients was not limited, allowing for a comprehensive assessment of the disease across different age groups.

In Ukraine, leptospirosis is a notifiable disease as mandated by the Ministry of Health order dated July 30, 2020, No. 1726. When a case is identified, it must be reported to local epidemiological departments within two hours via telephone. An emergency notification is then prepared, with a paper copy delivered within 18 hours, following the Ministry of Health order dated July 30, 2020 (No. 1726). Medical professionals and other healthcare workers who receive health-related information are responsible for completing these notifications.

Medical staff gather comprehensive patient histories, including potential exposure to contaminated water, animals, or recent travel. According to the Ministry of Health order No. 905 of December 28, 2015, leptospirosis diagnosis criteria include fever or at least two of the following symptoms: chills, headache, muscle pain, conjunctival redness, skin or mucous membrane hemorrhages, rash, jaundice, myocarditis, meningitis, kidney failure, or respiratory complications such as hemoptysis. However, clinical and epidemiological data alone are insufficient for confirmation, necessitating PCR or MAT.

Blood samples from suspected cases are analyzed using the MAT test at the Especially Dangerous Infections (EDI) laboratories. Initially, samples are tested at dilutions of 1:5 and 1:50. If positive, further dilutions (1:10, 1:100, 1:200, etc.) are performed according to the Methodological Recommendations 9.1.1.098-02 for Anti-Epidemic Measures and Laboratory Diagnostics of Leptospirosis (Goris and Hartskeerl, 2015). Control culture diluted 1:2 in phosphate-buffered saline is used to validate results, defining the endpoint as the serum dilution with 50% agglutination. Results are reported as endpoint dilution or titer, with antibody titers of ≥1:100 plus clinical and epidemiological data indicating infection. A fourfold increase in antibody titers between paired samples strongly suggests acute infection. MAT testing involves 14 *Leptospira* spp. serovars per local protocols (Methodological Recommendations 9.1.1.098-02, 2002). For patients who died with clinical leptospirosis, PCR analysis of kidney tissue sections using *lipL32* gene primers confirms the disease (Ahmed et al., 2020; Jayasundara et al., 2021).

Statistical analysis was performed using Jamovi version 2.2.5. Categorical variables were compared using χ² (Chi-square) tests to assess the association between various clinical characteristics and severe outcomes of leptospirosis. For each significant association identified, adjusted odds ratios (OR) were calculated to measure the strength of association between potential predictors and severe disease, along with their corresponding 95% confidence intervals (CI) to provide a range of uncertainty around the OR estimates. A Mann-Whitney U test was performed to compare the urea and serum creatinine levels between survivors and non-survivors of leptospirosis. Significance for all statistical tests was established at P < 0.05. Ethical approval for the study was granted by the Ethics Committee of Transcarpathian Regional Clinical Infectious Hospital.

## 3 Results

### 3.1 Leptospiral clinical signs as predictors of severe leptospirosis

**Figure 1:**
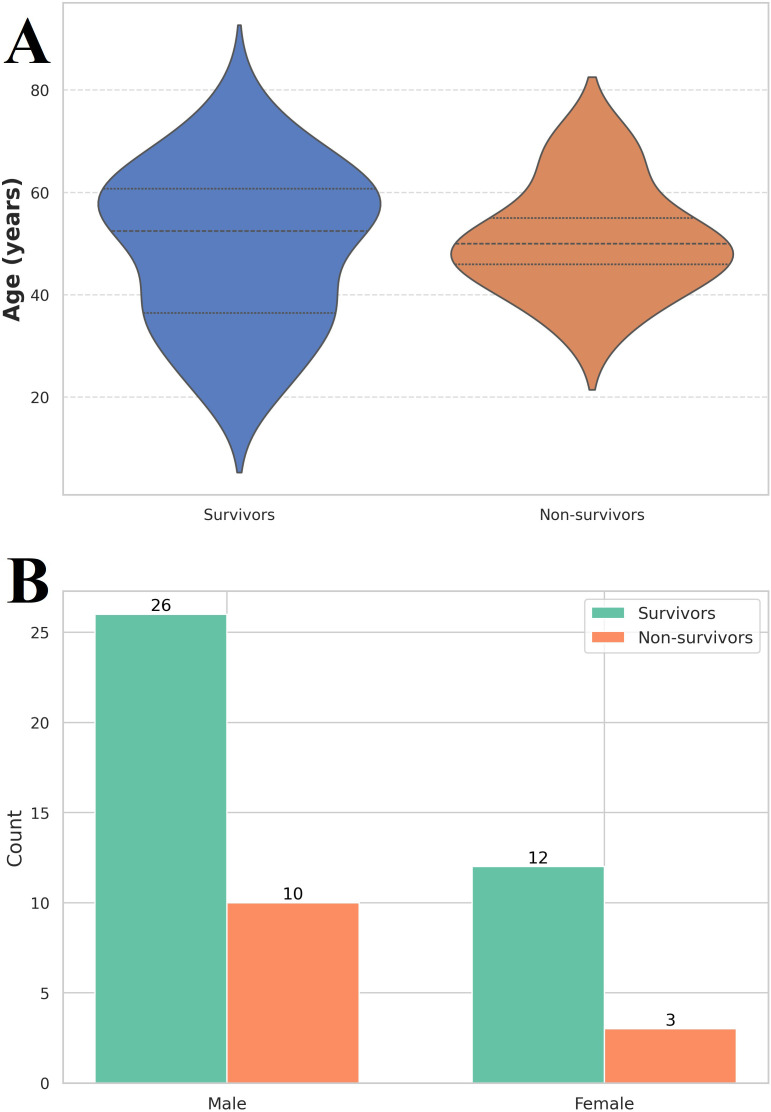
**(A)** Violin plot of age distribution among the investigated group. Survivors (n = 38) had a median age of 49 (36 – 61) years, while non-survivors (n = 13) had a median age of 50 (44 – 58) years. Label Y axe Age, years **(B)** Gender distribution among the investigated group. The vast majority of the 38 out of 51 patients were male (26 in the survivor’s group and 10 in the non-survivor’s group).

Out of the 51 patients involved in the study, 38 were categorized in the survivors’ group, while 13 were part of the non-survivors’ group. The majority were males, accounting for 70.5% of the cases, and the average age was 50 years (**Figures 1A, B**).

Upon admission to the hospital, the primary signs and symptoms observed in both groups were fever (78.4%), myalgia (76.4%), and jaundice (86.2%).

**Figure 2:**
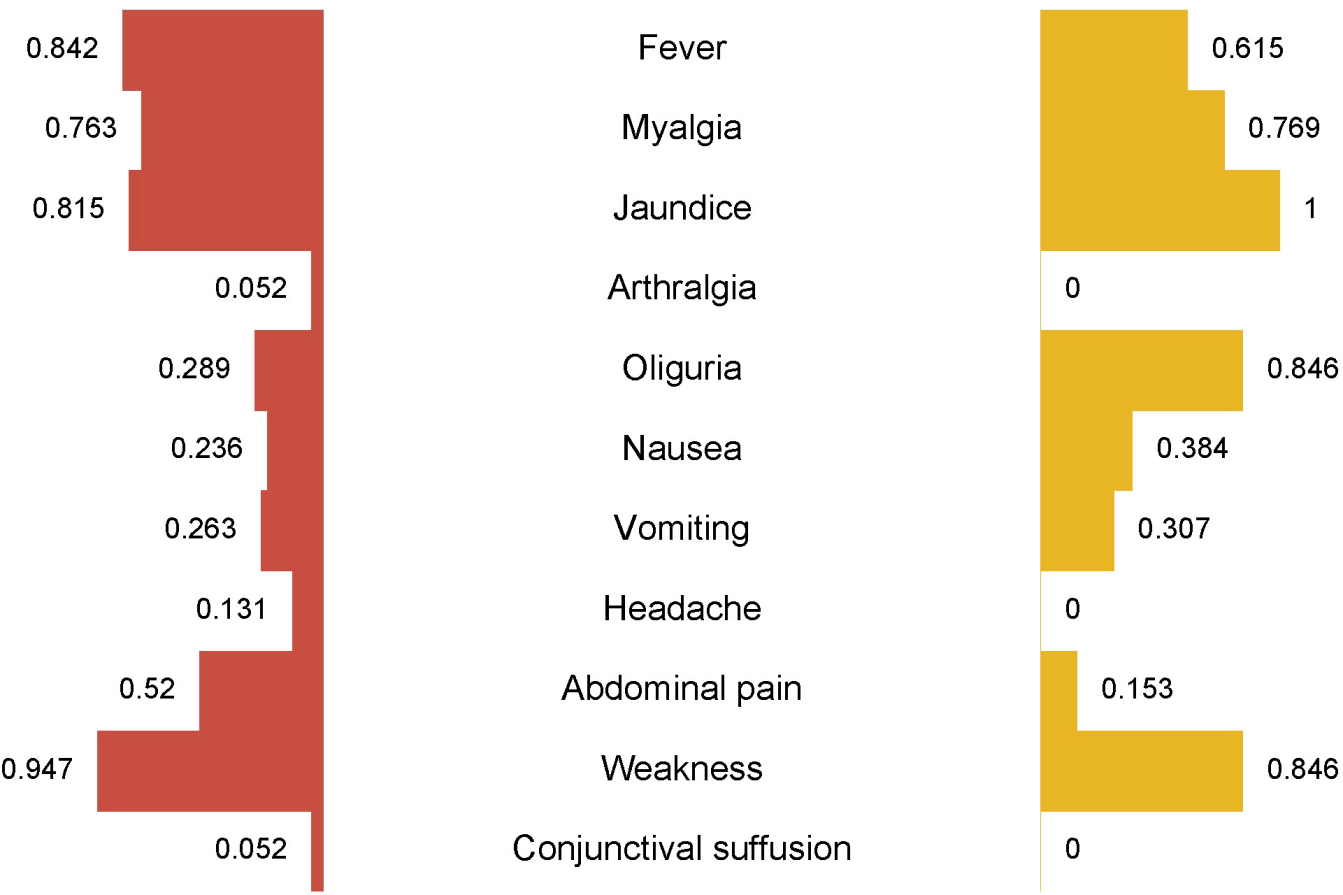
Analysis of clinical signs in patients with leptospirosis (butterfly bar plot). On the left (depicted in red), the frequencies of clinical symptoms are shown for the survivor’s group, while on the right (depicted in orange), the frequencies are shown for the non-survivor’s group. Oliguria was observed in 84.6% of patients with severe leptospirosis who died, compared to 28.9% in the survivor’s group.

All individuals with severe leptospirosis (n = 13, 100%) reported experiencing jaundice, and 11 patients exhibited oliguria (84.6%). Myalgia symptoms were complained of by 10 patients (76.9%), and 8 patients presented with fever (61.5%). Nausea was reported by 5 patients (38.4%), and abdominal symptoms were noted by 2 patients (15.3%) (**Figure 2**).

A chi-square test revealed a significant association between oliguria and severe outcomes, including mortality (p = 0.001). Oliguria was identified as the strongest predictor of severe leptospirosis, with an estimated odds ratio of 13.5, indicating a substantial risk increase for severe disease.

**Figure 3:**
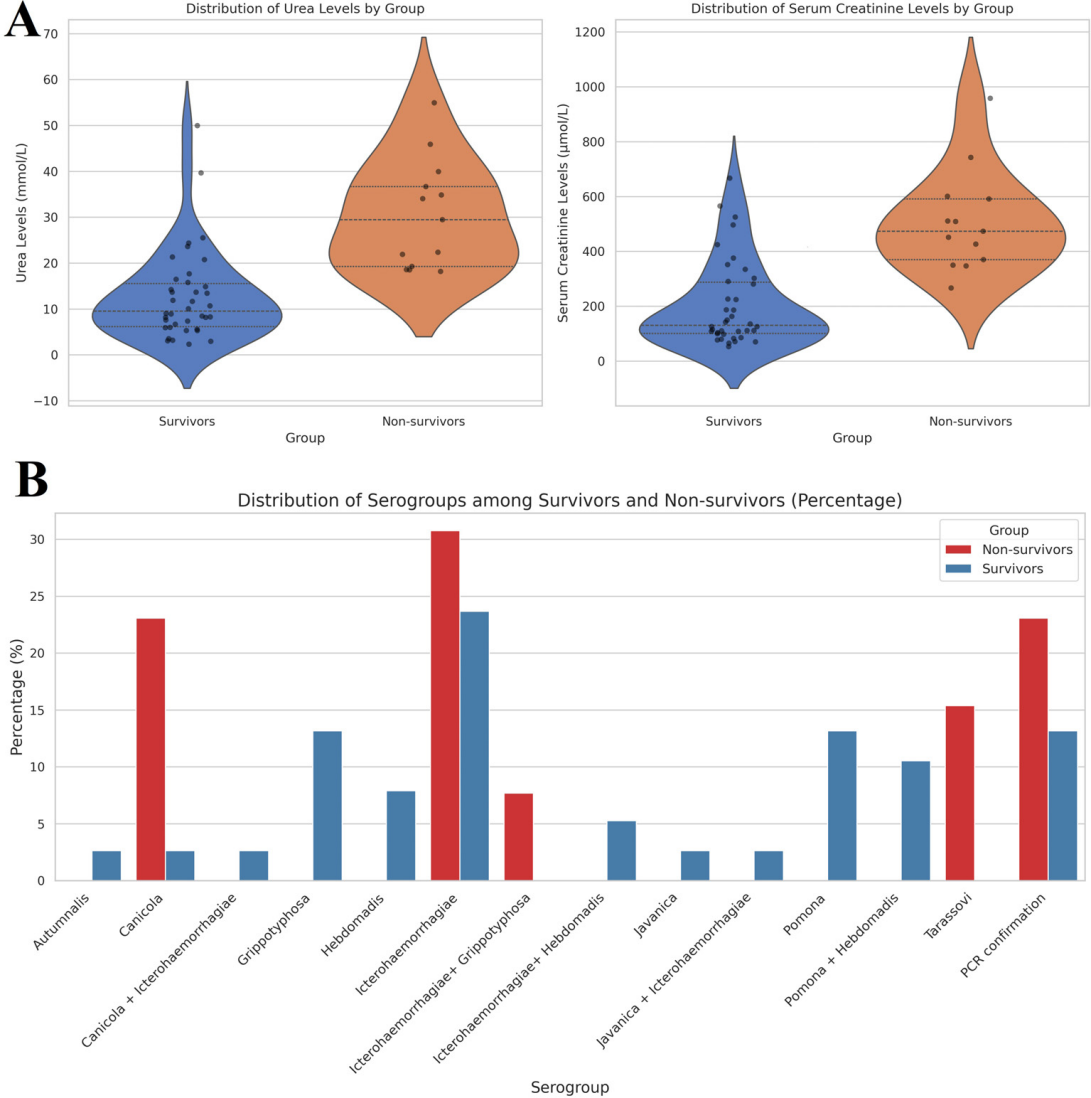
**(A)** Violin plot of distribution of serum creatine and urea levels among investigated groups. The median urea level among survivors was 10.2 mmol/L (interquartile range [IQR]: 7.4 - 15.7 mmol/L), whereas non-survivors exhibited a markedly elevated median level of 41.4 mmol/L (IQR: 34.0 - 55.0 mmol/L). Similarly, serum creatinine levels showed significant variation between the groups. Survivors had a median serum creatinine level of 112.3 µmol/L (IQR: 99.0 - 227.0 µmol/L), while non-survivors presented with a significantly higher median level of 510.0 µmol/L (IQR: 371.0 - 743.0 µmol/L). **(B)** Distribution of serogroups among investigated groups. Among non-survivors, Icterohaemorrhagiae was the most prevalent serogroup, accounting for 30.77%, followed by Canicola at 23.08% and Tarassovi at 15.38%. In contrast, survivors exhibited a higher prevalence of Grippotyphosa and Pomona, both at 13.16%. The analysis revealed that Icterohaemorrhagiae was significantly present in both groups, but it had a higher percentage in non-survivors (30.77%) compared to survivors (23.68%).

In our study, we also included an analysis of serum creatinine and urea levels upon admission. The analysis revealed significant differences in urea levels between the two groups. The median urea level among survivors was 10.2 mmol/L (interquartile range [IQR]: 7.4 - 15.7 mmol/L), whereas non-survivors exhibited a markedly elevated median level of 41.4 mmol/L (IQR: 34.0 - 55.0 mmol/L). Similarly, serum creatinine levels showed significant variation between the groups. Survivors had a median serum creatinine level of 112.3 µmol/L (IQR: 99.0 - 227.0 µmol/L), while non-survivors presented with a significantly higher median level of 510.0 µmol/L (IQR: 371.0 - 743.0 µmol/L). The analysis yielded a p-value of p < 0.001 (**Figure 3A**).

### 3.2 Distribution of Leptospira serogroups among survivors and non-survivors

The distribution of different serogroups was quantified as percentages for both survivors and non-survivors, as summarized in **Figure 3B**. Among non-survivors, Icterohaemorrhagiae (30.77%) was the most prevalent serogroup, followed by Canicola (23.08%) and Tarassovi (15.38%). In contrast, survivors showed a higher prevalence of Grippotyphosa (13.16%) and Pomona (13.16%). The analysis revealed that Icterohaemorrhagiae was significantly present in both groups but had a higher percentage in non-survivors (30.77%) compared to survivors (23.68%). Additionally, the presence of serogroups such as Grippotyphosa and Pomona was primarily observed in survivors, indicating a potential correlation between these serogroups and better clinical outcomes.

The results indicated a statistically significant difference in the distribution of serogroups between survivors and non-survivors, with a chi-square statistic of 22.60, a p-value of 0.0468, and 13 degrees of freedom. These findings suggest that the serogroups present in patients have an influence on disease severity and mortality.

### 3.3 Regional features of leptospirosis epidemiology

**Figure 4:**
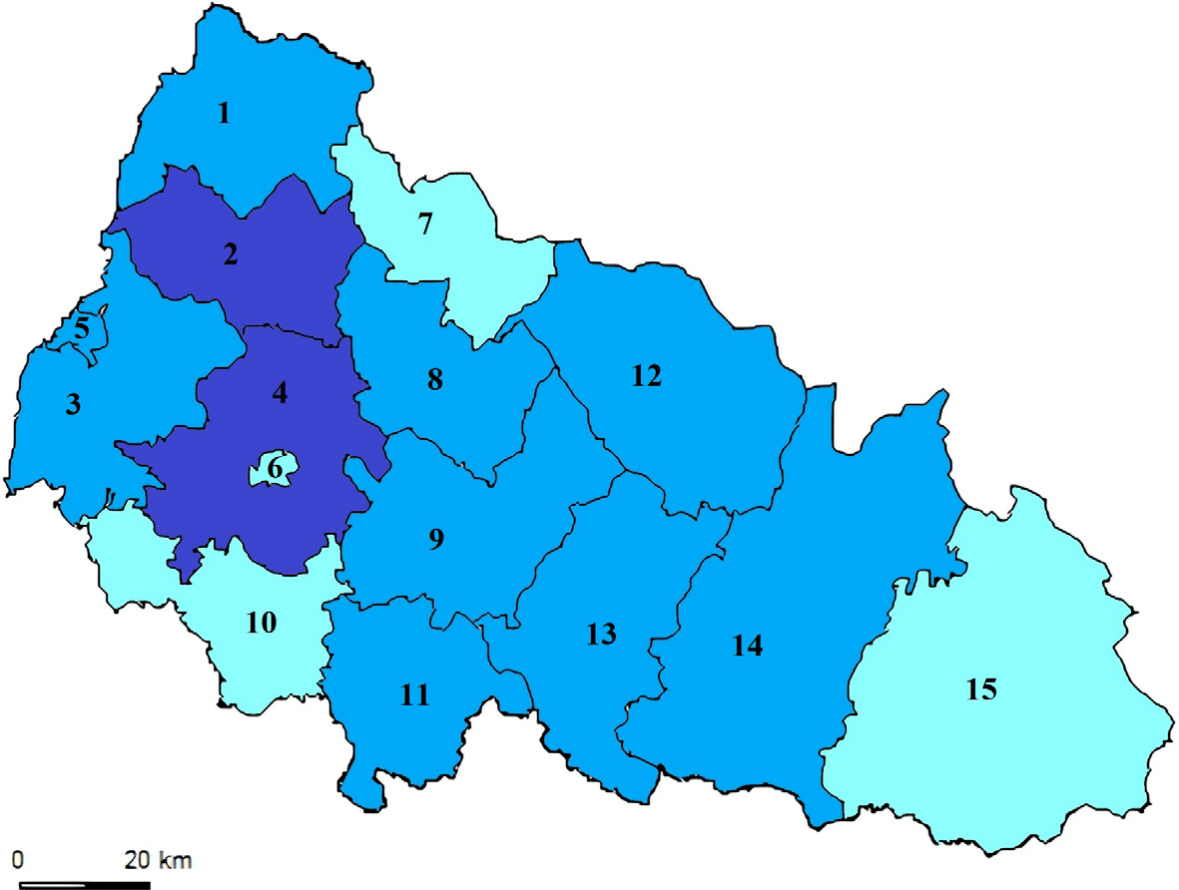
Leptospirosis Notification Rate (per 100K) in the Transcarpathian Region (2015-2022). The map illustrates the levels of leptospirosis incidence depicted in three color gradations. The blue districts indicate low notification rates (less than 0.72), which fall below the 25th percentile. Light blue represents districts with moderate notification rates, ranging between the 25th and 75th percentiles (specifically between 0.72 and 2.62). Dark blue signifies rates above 2.62, surpassing the 75th percentile. Districts and cities of the Transcarpathian region: 1. Velykobereznyansky District; 2. Perechyn District; 3. Uzhhorod District; 4. Mukachevo District; 5. Uzhhorod City; 6. Mukachevo City; 7. Volovetsky District; 8. Svalyavsky District; 9. Irshav District; 10. Berehiv District; 11. Vynogradivskyi District; 12. Mizhhirsky District; 13. Khust District; 14. Tyachiv District; 15. Rakhiv District.

As depicted in **Figure 2**, the relative number of patients in Transcarpathian region has consistently been higher. For the first time in 2023, it reached 12.08 per 100,000 population, surpassing the Ukrainian average by 12 times (**Figure 4**). We also analyzed the incidence of leptospirosis by district and found that the highest incidence was observed, in all years in Mukachevo District at 5.58 cases per 100,000 persons. This rate is four times higher than the Transcarpathian average. Additionally, Perechyn District had an incidence rate of 7.8 cases per 100,000 persons.

## 4 Discussion

This study aimed to identify predictive factors for lethality and severe leptospirosis in the Transcarpathian region.

Our analysis reveals a significant increase in the notification rate of leptospirosis in Ukraine in 2023. This surge is primarily attributed to the rising incidence of leptospirosis in Transcarpathia, accounting for 150 cases out of the total 433 in Ukraine, and the Ivano-Frankivsk region, contributing 34 cases.^1^

Zakarpattia oblast (also known as Transcarpathian region), located on the western border of Ukraine, shares boundaries with Romania, Hungary, Poland, and Slovakia, while also bordering Ivano-Frankivsk and L’viv oblasts to the east. In comparison, the rates for Transcarpathian region are significantly higher than those in neighboring countries, with Romania at 0.34, Slovakia at 0.15, Hungary at 0.13, and Poland at 0.01 per 100,000 population (Beauté et al., 2024). Characterized by a moderate continental climate with ample moisture, and moderate summers and winters, Zakarpattia presents geographical, landscape, zoological, and parasitological conditions influencing various natural focal diseases, including leptospirosis. The region has above-average human population density, with 63% residing in rural areas. High leptospirosis incidence is often reported in areas with abundant surface freshwater. The dense river system in Zakarpattia, influenced by high humidity and mountain relief, experiences increased water levels, spring flooding, and occasional disasters, as witnessed in 2010 (Wasiński and Dutkiewicz, 2013).

Comparing the notification rate with EU countries, Ukraine’s leptospirosis incidence surpasses that of all EU nations. France and Slovenia reported the highest rates at 0.76 and 0.82, respectively (Beauté et al., 2024).

The findings suggest that oliguria serves as a risk factor for severe leptospirosis. Recognizing this sign upon admission allows for early identification and the management of diuresis can alert physicians to the potential development of severe leptospirosis, enabling prompt initiation of aggressive treatment. The kidneys are a primary target of Leptospira, with kidney damage observed in 20–85% of patients (Meneses et al., 2020). Renal failure, particularly in cases with oliguria, stands as a well-established predictor of death and is linked to more frequent pulmonary involvement (Bharti et al., 2003; Silva Junior et al., 2011).

Leptospirosis is frequently linked to acute kidney injury (AKI), characterized by acute interstitial nephritis and acute tubular necrosis (Chou et al., 2023). The clinical presentation of AKI in leptospirosis is notably marked by non-oliguric and hypokalemic conditions, observed in 41–45% of patients experiencing AKI related to leptospirosis (Seguro et al., 1990; Lin et al., 1999). Conversely, disseminated intravascular coagulation has been noted in patients with severe leptospirosis complications, with septic AKI often presenting oliguria and anuria, likely resulting from acute tubular necrosis (Chierakul et al., 2008). Several factors contribute to the pathogenesis of AKI in leptospirosis, including the direct nephrotoxic effects of Leptospira, immune responses induced by toxins, and secondary effects from dehydration and hypoxia (Rista et al., 2022).

The acute phase of leptospiral infection typically lasts about one week, followed by the development of symptoms five to fourteen days later, leading to an immunological phase (Brito Monteiro et al., 2021). Severe cases can trigger a cytokine storm, characterized by elevated levels of TNF-α, IL-6, and IL-10 in the bloodstream (Haake and Levett, 2015). A study conducted in Sri Lanka in 2021 indicated increased levels of monocyte chemoattractant protein (MCP)-1 and kidney injury molecule (KIM)-1 in the blood and urine of patients with AKI linked to leptospirosis, with KIM-1 showing greater specificity than MCP-1 (Nisansala et al., 2021).

In a mouse model of leptospiral infection during the acute phase, TNF-α, IL-6, IL-10, MCP-1, and KIM-1 exhibited high expression levels. Furthermore, notable increases in renal lipocalin 2 (LCN2, also known as neutrophil gelatinase-associated lipocalin [NGAL]) and IL-34 expression levels have been documented (Chou et al., 2018). Previous investigations have established a correlation between urinary NGAL levels and leptospirosis-associated AKI (Srisawat et al., 2015).

The data imply that risk stratification for leptospirosis patients should not solely rely on the presence of icterus. Jaundice results from damage to hepatic capillary vessels without hepatocellular necrosis. Retrospective studies both confirm (Durmaz Cetin et al., 2004; Daher et al., 2009), and contradict (Spichler et al., 2008; Daher et al., 2015) the role of jaundice as a predictor of death in this infectious disease.

Myalgia, commonly affecting the back and legs, is a frequent symptom of leptospirosis (Vijayachari et al., 2008). Most studies indicate that myalgia is not linked to mortality in cases of leptospirosis (Al-shere et al., 2012; Daher et al., 2015). Histologically, there is evidence of focal necrosis of muscle fibers, often accompanied by a modest elevation in creatinine kinase (CK) (Lim, 2011). A study conducted by Dupont et al. in France reported higher CK levels in patients with leptospirosis who did not survive (Dupont et al., 1997).

Headache is a frequently observed symptom in cases of leptospirosis, often characterized by its severity and association with vomiting (Bharti et al., 2003). While impaired consciousness in the initial stages may progress to meningitic symptoms in approximately a quarter of cases during the immunological phase, it is noteworthy that headache was not a prevalent presentation in this particular series.

To improve the generalizability of these findings, future studies should incorporate a broader range of geographical regions with varying epidemiological characteristics of leptospirosis. Conducting multicentric prospective studies, ideally with a larger sample size, could better account for the role of environmental and socioeconomic factors in disease progression. In addition, future research should aim to control for potential confounding variables, such as pre-existing conditions, immunocompromised status, and co-infections, to provide more precise insights into clinical predictors of severe leptospirosis.

Biomarkers such as kidney injury molecule-1 (KIM-1) and neutrophil gelatinase-associated lipocalin (NGAL), which have shown promise in other studies, could also be incorporated into future research to enhance the prediction of severe outcomes. Studies focused on the immunological mechanisms, particularly the cytokine storm phenomenon, may help identify early intervention strategies to prevent the progression to severe or fatal illness.

## 5 Limitations

First, the retrospective design relies on existing medical records, which can lead to potential biases due to incomplete or inconsistent data. In particular, the diagnosis of leptospirosis was based on clinical presentation and laboratory confirmation using MAT or PCR, but co-infections or other underlying health conditions that could have influenced the severity of the disease were not systematically recorded. For example, common co-infections such as dengue or malaria, particularly in regions with similar environmental conditions, may complicate the clinical picture and affect patient outcomes.

Additionally, while this study focused on the Transcarpathian region, external factors such as regional variations in healthcare quality, availability of early intervention, and environmental conditions unique to the region could limit the generalizability of our findings. The study region has high humidity, frequent flooding, and proximity to natural reservoirs, factors that may contribute to a higher incidence and severity of leptospirosis compared to other regions. Thus, further research is necessary to explore whether the identified clinical predictors, such as oliguria, hold similar significance in lower-incidence areas with different environmental and healthcare contexts.

## Data Availability

The raw data supporting the conclusions of this article will be made available by the authors, without undue reservation.

## Footnotes

1 Available online at: https://phc.org.ua/kontrol-zakhvoryuvan/inshi-infekciynizakhvoryuvannya/infekciyna-zakhvoryuvanist-naselennya-ukraini. (Accessed June 23, 2024).
